# Long-term outcome of displaced radial neck fractures in adulthood: 16–21 year follow-up of 5 patients treated with radial head excision

**DOI:** 10.3109/17453670902967307

**Published:** 2009-06-01

**Authors:** Magnus K Karlsson, Pär Herbertsson, Anders Nordqvist, Ralph Hasserius, Jack Besjakov, Per Olof Josefsson

**Affiliations:** ^1^Department of Clinical Sciences, Lund UniversityMalmöSweden; ^2^Departments of Orthopedics Radiology, Malmö University HospitalMalmöSweden

## Abstract

**Background** There have been no reports on the long-term outcome of radial neck Mason type IIIb fractures in adults.

**Methods** 3 women and 2 men, aged 46 (22–69) years when they sustained a radial neck Mason type IIIb fracture, were evaluated after an average of 18 (16–21) years. All had been treated with radial head excision.

**Results** 3 individuals had no subjective elbow complaints while 2 reported occasional weakness. None had severe elbow complaints. The maximum elbow-to-elbow difference in range of motion was a deficit of mean 10° in extension in the injured elbow. Mean deficits in elbow flexion, forearm pronation, and forearm supination were below 5° and the mean difference in cubitus valgus angle was only 2° . There was no instability and no recurrent elbow dislocations. Radiographically, there were cysts, sclerosis, and osteophytes in all formerly injured elbows but none in the uninjured elbows. We found reduced joint space in 1 elbow that had been formerly injured.

**Interpretation** Mason type IIIb fracture in adults, treated with radial head excision, appears to have a favorable long-term outcome.

## Introduction

Minor displaced radial head and neck fractures in adults are reported to have a favorable outcome (Herbertsson et al. 2005) while displaced and comminuted fractures are often reported to have an inferior outcome (Arner et al. 1957, Herbertsson et al. 2004, Struijs et al. 2007, Rosenblatt et al. 2008). However, conflicting results have been reported (Mason 1954, Arner et al. 1957, Broberg 1986, Herbertsson 2004, Herbertsson et al. 2004a, Struijs et al. 2007, Rosenblatt et al. 2008). One reason for this could be that most reports have mixed displaced 2-fragment radial head fractures (type IIa), comminuted radius head fractures (type IIIa), minor displaced radial neck fractures (type IIb), and displaced radial neck fractures (type IIIb) in the evaluations. This may be erroneous, as comminuted fractures—which are often associated with a high-energy trauma (Herbertsson 2004, Herbertsson et al. 2004b)—may have inferior outcome compared to 2-fragment fractures. The few studies that have investigated Mason type IIIb fractures in adults include case reports dealing with nonunion (Karpinski 1982, Faber et al. 1995, Inhofe and Moneim 1998) or surgical techniques (Metaizeau et al. 1993, Keller et al. 1994, Smith et al. 2007) but not long-term outcome. We therefore report the long-term outcome of radial neck Mason type IIIb fractures in 5 adults, all of which were treated with radial head excision.

## Patients and methods

Using the radiographic archives at our hospital, where all radiographs have been saved for half a century, we scrutinized all elbow fractures from 1969–1979. We found 756 individuals with an isolated radial head or neck fracture without an associated elbow dislocation. Only 5 adults were reported to have a Mason type IIIb fracture of the radial neck: 3 women and 2 men with a mean age of 46 (22–69) years at injury. 1 injury was the result of a fall from a height of > 2 meters and 4 injuries were the result of low-energy trauma, defined as a fall of < 2 meters or direct impact. None had had other major fractures or soft-tissue injuries such as elbow dislocations, and all had been treated with a radial head excision. No complications were recorded during or after surgery.

The subjective outcome was assessed by a questionnaire that evaluated activities of daily living (ADL), elbow pain on loading and at rest, tenderness, range of motion, stability, and strength in the affected elbow. Mobility and strength in the wrist and the hand were also evaluated. The uninjured arm served as a control in all comparisons. The objective outcome was measured with a goniometer and included flexion and extension of the elbow and wrist, pronation and supination of the forearm, and the angle of the extended elbow. The grip strength of the hand was measured with a Martin vigorimeter. The circumference of the arm and forearm was measured with a tape 10 cm distal and proximal to the tip of the olecranon. The uninjured arm served as a control.

**Table:** Range of motion in elbows and wrists, circumference of upper arm and forearm 10 cm from the olecranon tip, and grip strength in the arms of 5 individuals with an isolated Mason type IIIb fracture of the radial neck 18 years on average after the injury. Data presented are mean (range)

	Formerly fractured arm	Non–fractured arm
Elbow flexion (°)	139 (125–145)	141 (135–145)
Elbow extension (°)	–6 (–15–0)	4 (0–10)
Forearm pronation (°)	80 (70–90)	83 (70–90)
Forearm supination (°)	88 (80–90)	88 (80–90)
Elbow valgus angle (°)	12 (10–15)	10 (5–10)
Wrist flexion (°)	71 (60–85)	73 (65–85)
Wrist extension (°)	65 (60–75)	66 (60–75)
Circumference, upper arm (cm)	27 (24–29)	26 (23–28)
Circumference, forearm (cm)	24 (22–27)	23 (22–26)
Grip strength (kp/cm^2^)	0.6 (0.3–0.7)	0.6 (0.5–0–7)

On the basis of the primary radiographs, the fractures were classified according to the Mason classification modified by Broberg and Morrey (Mason 1954, Broberg 1986). The evaluation was done by a radiologist with no knowledge of the treatment, or of the subjective or clinical outcome of the patients. Follow-up radiographs included anterior-posterior and lateral projections of both elbows. Subchondral cysts, subchondral sclerosis, and/or osteophytes were defined as degenerative changes, and any more than a 1-mm reduction in the medial joint space was recorded.

The ethics committee of Lund University, Sweden, approved the study (LU 343-95), which was carried out in accordance with the Helsinki Declaration of 1975.

## Results

3 patients rated their formerly fractured elbows to be without problems (i.e. with no subjective complaints) and 2 rated their elbows as having slight impairment, mostly occasional weakness. None reported severe impairment such as pain at rest, tenderness, or constant weakness. The mean deficit in elbow extension was 10°. The mean deficit in elbow flexion, forearm pronation, and forearm supination were all below 5° (Table). 1 individual had an elbow extension deficit of 25°; in all other individuals the deficits in extension, flexion, supination, and pronation were 10° or less. None had a cubitus valgus angle exceeding 5°. Radiographs showed cysts, sclerosis, and osteophytes in all formerly injured elbows but not in the uninjured elbows. Reduced medial joint space was found in 1 formerly injured elbow.

## Discussion

We found that 18 years after a Mason type IIIb fracture in adulthood on average, 5/5 former patients rated their previously injured elbow as having no or only minor disability after treatment with a radial head excision. Only 2 papers have reported the long-term outcome of Mason type IIIb fractures in adults (Herbertsson et al. 2004a,b), and both reports mixed Mason type IIIa (radial head) and type IIIb (radial neck) fractures. The reason that there have been so few publications is that this type of fracture is uncommon in adults; we found 5 patients with Mason type IIIb in a material of 756 patients who sustained radial head and neck fractures.

One complication that could arise after a proximal radial fracture treated with radial head excision is cubitus valgus. Mikic and Vukadinovic (1983) reported that this gradually develops in patients treated with radial head excision, a notion also supported by Herbertsson et al. (2004a). In contrast, none of our 5 individuals developed a cubitus valgus exceeding 5°.

The high proportion of degenerative changes in the formerly injured elbows supports the results of previous publications reporting degenerative changes in three-quarters of adults who have had a radial head Mason type II or III fracture (Mason 1954, Broberg 1986), a proximal radius fracture treated with a radial head excision (Mason 1954, Broberg 1986), or a radial head or neck fracture treated with a radial head excision (Herbertsson et al. 2004a). Previous long-term studies have, however, shown that these radiographic deformities are of minor clinical relevance (Mason 1954, Broberg 1986, Herbertsson et al. 2004a), a view supported by the current report.

The clinical outcome presented in this study appears to be similar to the outcome following Mason type IIb fractures of the radial neck in adults. Arner et al. (1957) reported that 95% of patients with a Mason type II fracture had no complaints 1–15 years after the injury and Poulsen et al. (1974) reported that 7 of 7 patients with nonoperatively treated Mason type IIb fractures had excellent outcome 5 years on average after the injury.
